# Progress of Multiparameter Magnetic Resonance Imaging in Bladder Cancer: A Comprehensive Literature Review

**DOI:** 10.3390/diagnostics14040442

**Published:** 2024-02-17

**Authors:** Kangwen He, Xiaoyan Meng, Yanchun Wang, Cui Feng, Zheng Liu, Zhen Li, Yonghua Niu

**Affiliations:** 1 Department of Radiology, Tongji Hospital, Tongji Medical College, Huazhong University of Science and Technology, Wuhan 430030, Chinaxymeng@hust.edu.cn (X.M.); zhenli@hust.edu.cn (Z.L.); 2 Department of Urology, Tongji Hospital, Tongji Medical College, Huazhong University of Science and Technology, Wuhan 430030, China; 3 Department of Pediatric Surgery, Tongji Hospital, Tongji Medical College, Huazhong University of Science and Technology, Wuhan 430030, China

**Keywords:** bladder cancer, multiparameter magnetic resonance imaging (mpMRI), functional sequences, Vesical Imaging-Reporting and Data System (VI-RADS), artificial intelligence (AI)

## Abstract

Magnetic resonance imaging (MRI) has been proven to be an indispensable imaging method in bladder cancer, and it can accurately identify muscular invasion of bladder cancer. Multiparameter MRI is a promising tool widely used for preoperative staging evaluation of bladder cancer. Vesical Imaging-Reporting and Data System (VI-RADS) scoring has proven to be a reliable tool for local staging of bladder cancer with high accuracy in preoperative staging, but VI-RADS still faces challenges and needs further improvement. Artificial intelligence (AI) holds great promise in improving the accuracy of diagnosis and predicting the prognosis of bladder cancer. Automated machine learning techniques based on radiomics features derived from MRI have been utilized in bladder cancer diagnosis and have demonstrated promising potential for practical implementation. Future work should focus on conducting more prospective, multicenter studies to validate the additional value of quantitative studies and optimize prediction models by combining other biomarkers, such as urine and serum biomarkers. This review assesses the value of multiparameter MRI in the accurate evaluation of muscular invasion of bladder cancer, as well as the current status and progress of its application in the evaluation of efficacy and prognosis.

## 1. Introduction

Bladder cancer (BC) is the tenth most common cancer in the world, with nearly 573,000 new cases and 213,000 deaths worldwide each year [1]. The global age-standardized incidence per 100,000 population per year is 9.5 for males and 2.4 for females. In the European Union, it is 20 for males and 4.6 for females [2]. The standardized mortality rate per 100,000 population per year is 3.3 for males and 0.86 for females [2]. The overall morbidity and mortality are increasing year by year. Multiple factors contribute to the development of bladder cancer, with smoking being the most important risk factor, followed by occupational exposure to chemicals such as aromatic amines or chlorinated hydrocarbons, infection with blood schistosomiasis parasites, and exposure to ionizing radiation [2].

Bladder cancer is a urothelial tumor originating from the mucosa membrane of the bladder. Approximately 75% of cases are urothelial carcinoma, and about 25% belong to different histological types [3], which adds to the complexity of bladder cancer management. Based on standardized histo-morphological characteristics described by the World Health Organization, bladder cancer can be classified into low-and high-grade urothelial carcinomas [4]. Based on tumor invasion depth, it can be categorized as non-muscle-invasive bladder cancer (NMIBC) or muscle-invasive bladder cancer (MIBC) [5]. About 70% of cases are confined to the mucosa (Ta stage or carcinoma in situ) or submucosa (T1 stage), of which about half involve Ta tumors. NMIBC usually has a relatively slow natural course of disease, and treatment aims at reducing local recurrence and staging progression while maintaining patients’ quality of life. Transurethral resection of bladder tumor (TURBt) is a common treatment method for NMIBC, followed by appropriate intravesical instillation chemotherapy or systemic chemotherapy postoperatively [6]. About 30% of cases invade the muscularis propria and are classified as MIBC, with a poor prognosis based on the primary tumor stage and regional lymph node status. Common treatment modalities include radical cystectomy, TURBt, and systemic chemotherapy alone or in combination with other methods [7,8]. Due to the influence of operator experience, about 25% of patents may be staging-inaccurate. Pathologists may also show inconsistencies of 10–29% when grading urothelial cancers [9], and the staging inconsistency may range from 15 to 56% [10]. These factors can significantly influence treatment decisions for patients. The treatment and prognosis of bladder cancer are closely related to accurate preoperative diagnosis, and the current diagnostic methods still have certain limitations and deficiencies.

Magnetic resonance imaging (MRI) is currently the most accurate non-invasive imaging method to identify muscular invasion of bladder cancer; it can achieve preoperative staging and grading evaluation of tumors, but there are still certain limitations, such as the relatively poor accuracy of lymph node staging [11,12]. The purpose of this review is to summarize the value of quantitative and qualitative multiparameter MRI (mpMRI) in the accurate assessment of muscular invasion of bladder cancer before surgery, and the current status and progress of its application in the evaluation of efficacy and prognosis. The literature was retrieved from the PubMed and Web of Science databases, the keywords were bladder cancer and magnetic resonance imaging, and the selected period was nearly 10 years. The literature was screened and summarized based on diffusion-weighted imaging, dynamic contrast enhancement, mpMRI, VI-RADS, radiomics, and various combinations mentioned in the Introduction section. The literature language was limited to English, and the literature formats included articles, reviews, and meta-analyses. This work first reviewed the application of single MR sequences, mainly including diffusion-weighted imaging (DWI) and dynamic contrast-enhanced (DCE), followed by the combination of sequences and the most commonly used Vesical Imaging-Reporting and Data System (VI-RADS), focusing on the application of quantitative parameters in the staging and grading of bladder cancer, as well as tumor invasiveness, treatment effect, and prognosis. At the same time, the clinical application status and prospect of VI-RADS were reviewed. This comprehensive review of current MR research on bladder cancer is intended to help both radiologists and urologists optimize treatment and management strategies, and better achieve individualized and accurate management of patients.

## 2. The Role of DWI in Bladder Cancer

DWI is a functional MRI technique that measures the diffusion of water molecules. It provides non-invasive insights into tissue physiology, using contrast derived from variations in the Brownian motion of water molecules. DWI can serve as a biomarker to quantitatively reflect the pathological and physiological information of tumors [13,14].

### 2.1. The Role of DWI in Staging and Grading of Bladder Cancer

Since Matsuki et al. first applied DWI to the diagnosis of bladder cancer [15], numerous studies have demonstrated its powerful diagnostic efficacy [13,16,17]. When the b-value is between 800 and 1000 s/mm^2^, there is an obvious signal contrast between bladder cancer and surrounding tissues, maintaining a high image quality [18]. The apparent diffusion coefficient (ADC) is used to quantify the degree of diffusion of water molecules which influences cell structural complexity and reduces extracellular space. Numerous studies have found a correlation between ADC value and the histological grade of bladder cancer [19]. Notably, ADC values for high-grade bladder cancer were significantly lower than those for low-grade bladder cancer, reflecting changes in tissue biological characteristics and microstructure changes. The Receiver Operating Characteristic (ROC) curve, based on ADC value, displays excellent diagnostic efficacy for predicting invasive bladder cancer (AUC = 0.921) [20]. Certain DWI data processing models, such as the Fractional-order Calculus (FROC) model, can improve diagnostic efficacy. The model can distinguish NMIBC from MIBC, as well as high-grade and low-grade cancers. The diagnostic efficiency of parameters D (AUC = 0.842) and μ (AUC = 0.857) obtained by the FROC model is higher than that of the conventional mono-exponential model (AUC = 0.804) [21].

Secondary fibrosis and inflammation of the bladder wall often occur after TURBt. This results in irregular bladder wall thickening, which is challenging to differentiate from tumor residue. DWI offers high diagnostic efficiency in distinguishing tumor residue from postoperative reactions, with an accuracy rate of 87% [22]. This is superior to T2-weighted imaging (T2WI) and DCE sequences. DWI can enhance the evaluation of disease status post-TURBt, accurately identify postoperative tumor residue, and facilitate appropriate reoperations. MpMRI has considerable sensitivity and specificity in detecting MIBC after TURBt and other treatments. However, it is less sensitive when it comes to infiltration of peripheral fat and involvement of lymph nodes [23]. Therefore, it may play a significant role in postoperative local staging. However, ADC values can be influenced by scanning instrument or parameters. Toshinori Nishizawa, for the first time, used the ADC value of the gluteus maximus to internally standardize the ADC value of bladder cancers [24]. That study concluded that the method effectively eliminated the discrepancy in ADC values between different protocols, providing a reliable way to evaluate different scanning protocols for predicting the invasive phenotype of bladder cancer.

### 2.2. The Role of DWI in the Evaluation of Biological Invasiveness and Prognosis of Bladder Cancer

DWI is highly valuable for evaluating the biological invasiveness and prognosis of bladder cancer. Ki-67, a cell proliferation marker, has a labeling index (LI) that correlates with recurrence, progression, and patient survival rates in bladder cancer [17]. Ki-67 LI tends to be higher in sessile, larger, higher-grade, or higher-T-stage bladder tumors and is negatively correlated with the ADC value. The ADC value can indicate the invasion and proliferation ability of bladder cancer, making it a potential biomarker for invasiveness. This correlation is also linked to patient survival, aiding in clinical decision making [25]. In a small retrospective study involving 17 cases of aggressive high-grade bladder cancers, Rosenkrantz et al. reported an association between ADC values and metastasis, with metastasis bladder cancers having significantly lower ADC values than non-metastatic bladder cancers [26]. ADC values are also correlated with the histological and biological aggressiveness of bladder cancer. Funatsu et al. found that bladder cancer with low ADC values had a high recurrence rate, underlining its usefulness in clinical decision making [27]. Sevcenco et al. used an artificial neuron network (ANN) combined with ADC values and the disease-specific survival rate (DSS) to build a combination model [28]. Their study showed that ADC values can predict DSS in patients with bladder cancer with high accuracy and can identify patients at high risk of disease-related death. Bladder cancers exhibit diverse morphological manifestations after neoadjuvant chemotherapy (NAC), including a completely normal appearance, nodular enhancement, fibrosis, or inflammatory changes, particularly pathological complete response (pCR) [29]. The ADC value effectively diagnoses pCR after NAC in MIBC patients (AUC = 0.92) [30]. Zhang et al. compared the ADC values and ADC histogram parameters of the response group and the non-response group of MIBC patients after NAC [31]. Their results showed significant differences in parameters between the two groups, suggesting that ADC values and histogram parameters can be used to evaluate efficacy.

Tumor heterogeneity is an important indicator for differentiating benign and malignant bladder tumors, as well as being an effective indicator for evaluating treatment effect [32]. The quantitative values of tumor homogeneity obtained by ADC histogram analysis of the whole tumor can be used to evaluate tumor heterogeneity. The results of the referenced study showed that the spatial distribution of ADC values in the NAC response group was more heterogeneous. The application of ADC histogram can predict chemotherapy response before treatment and improve the prognosis of patients [33]. Necchi et al. highlighted the role of MRI in assessing treatment response before and after immunotherapy, allowing non-invasive imaging prediction of pCR status by administering pembrolizumab to patients with stage T2-4aN0M0 bladder cancer prior to radical cystectomy [34].

### 2.3. Intravoxel Incoherent Motion (IVIM) and Diffusion Kurtosis Imaging (DKI)

As with ADCs, IVIM and DKI can also be used for histological evaluation of bladder cancer. Compared to conventional DWI, IVIM-DWI can reflect the correlation between tumor perfusion and true diffusion simultaneously, and hence more accurately reflect the true diffusion of tumors [35]. In IVIM, the combination of D and f parameters showed the highest diagnostic efficiency in assessing the aggressiveness of bladder cancer (AUC = 0.931), exceeding the diagnostic efficiency of a single parameter [36]. The parameters of biexponential and stretched exponential models assist in staging (AUC = 0.945, 0.912, 0.946) and grading (AUC = 0.866, 0.862, 0.856) of bladder cancer, and have high diagnostic efficiency [37]. The D value obtained by IVIM demonstrates superior diagnostic efficiency to that of traditional DWI in distinguishing NMIBC from MIBC, and it has a significant correlation with Ki-67 LI, which can be used as a potential biomarker to evaluate the aggressiveness of bladder cancer [38].

DKI originated from DWI, and various studies have confirmed that DKI plays an important role in pathological grade and tumor stage [39,40]. Kurtosis is superior to diffusion in differentiating MIBC from NMIBC. Directed kurtosis (Kr) may become a valuable biomarker for assessing the aggressiveness of bladder cancer [41]. Li et al. used the multi-b-value DWI sequence, the mono-exponential model, and the DKI model for data processing, comparing the value of two models in identifying muscular invasion of bladder cancer [42]. Their study showed that both the traditional mono-exponential model and the DKI model were helpful in distinguishing MIBC from NMIBC. The combination of DKI parameters (Dapp and Kapp) yielded a higher diagnostic value in assessing bladder cancer aggressiveness than the ADC values of the traditional mono-exponential model. Tumor Contact Length (TCL), the curve-like contact length between the bladder wall and the tumor, has been used to predict tumor blood vessels [43,44]. TCL displayed good diagnostic value for MIBC (AUC = 0.90), and the cutoff value was 3 ± 0.3 cm. Both Kapp and TCL were independent risk factors for MIBC, and their combination offered the highest diagnostic value (AUC = 0.95) [45,46].

### 2.4. The Role of DWI Radiomics in Bladder Cancer

Radiomics has gained attention in recent years due to its ability to extract and analyze quantitative features of DWI images. This transforms medical images into minable high-dimensional data to support decision making through quantitative analysis [47]. For example, multiparameter radiomic methods based on T2WI and ADC can effectively evaluate the pathological grade of bladder cancer. This method has a high diagnostic efficiency (AUC = 0.923) and can assist radiologists and urologists in distinguishing bladder cancer grades [48]. Radiomics of mpMRI has advantages in evaluating myometrial invasion [49,50]. In addition, the Random Forest (RF) model was superior to TURBt in identifying MIBC, and the sensitivity increased to 0.964 after combination [51]. DWI radiomic features combined with TURBt can improve the sensitivity and accuracy of identification of MIBC, providing a reliable basis for clinical decision making [52].

Notably, the application of radiomics extends from preoperative diagnosis and evaluation to the assessment of disease efficacy and prognosis. Xu et al. demonstrated that a nomogram based on radiomics and clinical predictors can effectively predict the risk of bladder cancer recurrence two years post-surgery, with about 80% accuracy in training and validation cohorts [53]. Radiomic signatures of DWI have been used to predict the progression-free survival (PFS) of MIBC. These features were significantly correlated with PFS, but not with clinicopathological factors, indicating their potential as biomarkers for assessing PFS in MIBC patients [54]. The average ADC value and kurtosis in the histogram parameters are conducive to the differentiation of benign and malignant bladder lesions, and the average ADC value is the most valuable parameter in differentiating the stage of bladder cancer [16,55]. The GLCM texture feature in an ADC map may become an effective biomarker for predicting the pathological grade of bladder cancer. It can evaluate the efficacy of treatment of MIBC patients after radiotherapy and chemotherapy, and the omics features of DWI can be used as imaging biomarkers to optimize treatment strategies [56].

Radiomic models combined with clinical factors are superior to conventional MRI and simple radiomic models in calibration and discrimination [57]. They can be used as a reliable and non-invasive aid for preoperative identification of MIBC and NMIBC. In order to improve the diagnostic efficiency and application value of radiomics, a trend is developing of incorporating clinical risk factors such as tumor size and TURBt into the prediction model [58]. Radiomics extends its clinical value to therapeutic effect evaluation. For instance, a radiomic nomogram integrating clinical T-stage and three single-model radiomic models has been developed to predict tumor response to NAC. This model showed a higher diagnostic efficiency (AUC = 0.973) than other combined radiomics models, indicating its potential as a non-invasive tool for predicting the response of MIBC patients to NAC [59].

### 2.5. Diagnostic Value of DWI Imaging Features in Bladder Cancer

The high signal intensity of bladder tumors on DWI can be used as an effective method to identify NMIBC. Certain details, such as some lesions with a hypointense pedicle and inner thickening, can also be helpful. Takeuchi and his team proposed the MR staging criteria for bladder cancer [19]. They used the presence of submucosal hyposensitivity as a diagnostic basis for NMIBC. Furthermore, the stalk of the pedunculated tumor, composed of edematous submucosa, fibrous tissue, and capillaries, appears as a low signal on DWI. This is a feature of NMIBC. Typically, a pedunculated papillary tumor presents a “C”-shaped hyperintensity area with a hypo signal pedicle or submucosal thickening, known as the “ inchworm” sign [19,60,61,62]. The absence of this sign in T1 bladder cancer is an independent risk factor for tumor progression [63]. Conventional morphological MR sequences have high soft tissue resolution. For instance, combining T2WI with DWI sequences improved the accuracy of bladder cancer muscle-invasion assessment from 79% to 96% [20,64]. By combining the T2 Spectral Attenuated Inversion Recovery (SPAIR), DWI, and DCE imaging methods, Yuan et al. achieved an accurate assessment of myometrial invasion (with an accuracy of 95.2%) [65]. They further explained the difference in imaging features between Ta and T1 tumors. They found that 93% of tumors with thin lesions and low signal pedicle were Ta stage, and rootless tumors were mostly T1 stage.

## 3. The Role of DCE in Bladder Cancer

DCE is a technique used to evaluate angiogenesis, where tumor enhancement reflects increased vessel density, average vessel diameter, and vessel wall permeability. Micro-vessel density in bladder cancer correlates with tumor grade, stage, and potential malignancy [66]. It serves as a valuable indicator of tumor angiogenic activity and the formation of new blood vessels. This non-invasive and fast technique has distinct advantages. When compared to the conventional T2WI sequence, the DCE sequence can enhance the agreement between observers and improve the localization of small tumors and those with a thickened bladder wall [67].

Zhou et al. compared the correlation between DWI, DCE, and tumor invasiveness [68]. They found a negative correlation between the invasiveness of bladder cancer and the clearance rate of ADC value and DCE. The combination of the two resulted in 95.7% accuracy, suggesting that ADC and DCE clearance rate can effectively evaluate the invasiveness of bladder cancer. The accuracy of ADC outperforms that of DCE semi-quantitative parameters. By measuring the relative enhancement value of the arterial phase and venous phase in DCE, the effectiveness of chemotherapy in MIBC can be evaluated [69]. A combination of these two parameters offers the best diagnosis for identifying the complete response of NAC in MIBC [30].

Quantitative parameters of DWI and DCE can evaluate the stage, grade, aggressiveness, and prognosis of bladder cancer to a certain extent. Both the post-processing model and the extraction of radiomics features are based on relatively large sample studies, with many factors affecting quantitative parameters and complex calculations which cannot be widely used in clinical practice. Therefore, diagnosis based on image features has been paid more and more attention by both radiologists and urologists.

## 4. The VI-RADS

In 2017, Panebianco et al. used MR imaging features of bladder cancer as described in the literature [70]. For example, the bladder wall on T2WI showed a continuous low signal, while the tumor on DWI displayed a high signal with low signal stalk or inner layer thickened. The diagnosis feature of NMIBC was complete continuous linear enhancement of submucosal tissue adjacent to the tumor. This was the first instance of these three sequences being combined to subjectively determine muscular invasion, and the results indicated a high combined diagnosis accuracy (AUC = 0.96). Zhang et al. conducted a meta-analysis of four bladder studies using mpMRI, which mainly relied on the imaging features of T2WI, DWI, and DCE [71]. The results suggested that mpMRI had effective diagnostic ability for bladder cancer (AUC = 0.946) and could be used for staging of bladder cancer. A study involving 20 patients who met the inclusion criteria found that mpMRI had high sensitivity and specificity in diagnosing MIBC, particularly when myometrial invasion was initially suspected [12]. Therefore, mpMRI combined with morphology has been proven to be an effective method for the detection and accurate staging of bladder cancers.

**Figure 1:**
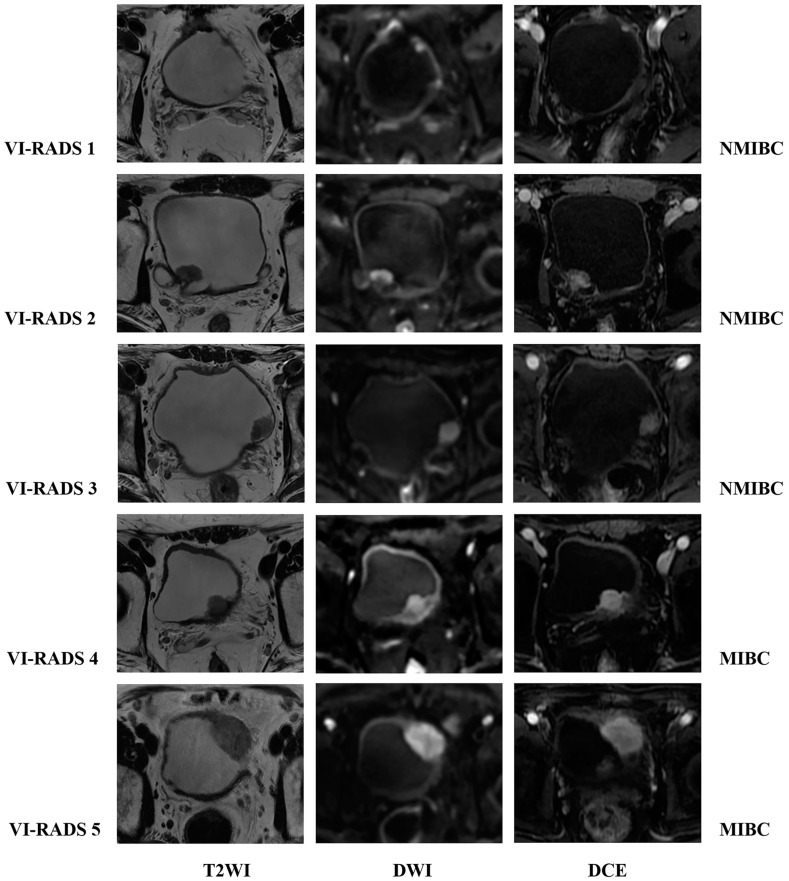
Schematic illustration of MRI appearances of Vesical Imaging-Reporting and Data System (VI-RADS) scores 1–5 using T2WI, DWI, and DCE; the pathological results were as follows: NMIBC, NMIBC, NMIBC, MIBC, and MIBC. Note: T2-weighted imaging, T2WI; diffusion-weighted imaging, DWI; dynamic contrast-enhanced, DCE; non-muscle-invasive bladder cancer, NMIBC; muscle-invasive bladder cancer, MIBC.

**Figure 2:**
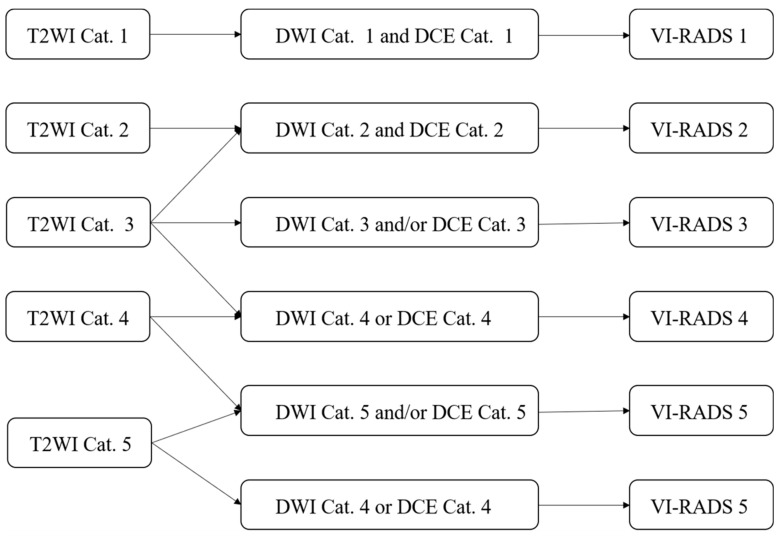
Flowchart of Vesical Imaging-Reporting and Data System (VI-RADS) overall scoring. Note: T2-weighted imaging, T2WI; diffusion-weighted imaging, DWI; dynamic contrast-enhanced, DCE; Category, Cat.

The VI-RADS was developed in 2018 by the European Association of Urology, the European Society of Urography, and the Japanese Society of Abdominal Radiology [10]. It aimed to standardize MR scanning protocols for bladder cancers, and provide a structured reporting system and a risk score for myometrial invasion (Figure 1 and Figure 2). The minimum image acquisition protocol includes high-resolution T2WI in three directions (axial, coronal, and sagittal) and two functional MRI techniques (DWI and DCE). High-field MRI (1.5T or 3.0T) scans are recommended for optimal spatial resolution and signal-to-noise ratio, while using a multi-channel phased array body coil of at least 16 channels. MRI spatial resolution can differentiate between the “inner” (mucosa and lamina propria), “outer” (muscularis propria), and periurical adipose tissue of the bladder. The muscle layer of the bladder showed a low signal on T2W images, and DWI and ADC showed a continuous moderate signal. On DCE sequence, the “inner layer” shows early enhancement, while the outer layer shows delayed or asymptotic enhancement. When there is muscle layer invasion, the muscle layer directly below the tumor will show early enhancement [72]. The consensus outlined in detail the scoring basis of T2WI, DWI, and DCE, and used the bladder diagram to locate the lesions. The final VI-RADS score is obtained according to the scoring process. The VI-RADS system diagnosis relies on the visual perception judgment of experts and is still a semi-qualitative diagnosis method. Its accuracy requires further testing, verification, and improvement.

### 4.1. The Value of VI-RADS in Evaluating Muscle Invasion in Bladder Cancer

At present, the VI-RADS scoring system has been validated by multiple research groups and has demonstrated good diagnostic performance in detecting muscle invasion of bladder cancer. Ueno et al. published the first validation study in 2019, which included 74 patients with bladder cancer and five readers for VI-RADS score [73]. The results showed excellent inter-reader agreement and good diagnostic performance for MIBC with an AUC of 0.90. This confirmed the accuracy and reproducibility of the system, although the sample size was relatively small. Wang et al. further validated the system with independent readings from 340 bladder cancer patients and two readers [74]. The study showed that when a cutoff value of ≥3 points or higher was used, the AUC for muscle invasion detection was 0.94, the sensitivity was 87.1%, and the specificity was 96.5%. These results provided further confirmation of the reproducibility and accuracy of the system in assessing muscle invasion in patients with untreated bladder cancer. However, the above studies did not compare the ability of individual sequences to predict muscle invasion. Each sequence is an essential component and cannot be replaced because T2WI and DCE may overestimate inflammatory changes, fibrosis, and edema around the tumor, while DWI can reduce excessive staging [75]. Further confirmatory studies are needed for lesions occurring at the bladder neck or bilateral ureteral opening, as well as images from MRI scanners with multiple magnetic field intensities.

Kim et al. retrospectively included 297 patients with bladder cancers and used cutoff values ≥3 and ≥4 to evaluate muscle invasion [76]. The results demonstrated that when the cutoff value was greater than 2, the accuracy of the VI-RADS scoring system was 63.7%, and when the cutoff value was greater than 3, the accuracy was 80.2%. The accuracy of the combined T2WI and DWI in detecting muscle invasion (79.3%) was similar to the overall VI-RADS score. Giudice et al. scored preoperative imaging data from 231 patients with bladder cancer and compared the VI-RADS scores from high-risk NMIBC (hr-NMIBC) patients with re-TURBt reports. The study showed that VI-RADS had good diagnostic performance in distinguishing between NMIBC and MIBC, with an AUC of 0.94 when the cutoff value was ≥3. In patients with hr-NMIBC, the AUC of differentiation between MIBCs predicted by VI-RADS was 0.93 [77], indicating the usefulness of re-TURBt stratification in patients with hr-NMIBC based on VI-RADS scores.

Multiple studies conducted up to 2020 have evaluated the diagnostic accuracy of VI-RADS for MIBC, using cutoff values of 3 or 4. These studies aimed to explore the impacts of different cutoff values on diagnostic accuracy and validate the performance of the scoring system [78]. Woo et al. conducted the first systematic review and meta-analysis of VI-RADS, including a total of six studies involving 1770 patients [79]. The overall diagnostic efficacy of VI-RADS showed an AUC of 0.94, a sensitivity of 0.83, and a specificity of 0.90. The study found that VI-RADS demonstrated good sensitivity and specificity in diagnosing MIBC. However, factors such as MRI scanner type and cutoff value should be taken into consideration as they may affect diagnostic performance due to significant associations with heterogeneity factors, such as patient numbers in each study, MRI field strength, T2WI layer thickness, and VI-RADS cutoff value (≥3 or ≥4). It is important to note that the study did not provide a detailed explanation of the impact of the cutoff value on diagnostic performance. In another study by Luo et al., a total of 1064 patients with bladder cancer were included in six studies [80], and when the cutoff value was 3, it was found that the combined AUC was 0.93, with sensitivity and specificity of 0.90 and 0.86, respectively. When using a cutoff value of 4, the combined AUC was approximately 0.92, with sensitivity of 0.77 and specificity of 0.97. The results indicated that VI-RADS has high sensitivity and specificity in predicting muscle invasion in bladder cancer, which is consistent with the results of previous studies. In addition, when a cutoff value of 3 or 4 was used, VI-RADS demonstrated similar diagnostic efficacy in detecting muscle invasion. Therefore, the selective utilization of the material application should be considered. MpMRI has become an important method for staging T-stage bladder cancer [81,82]. Defining the specific features of each sequence is crucial, and the application of appropriate MRI examination protocols preoperatively plays a crucial role in developing treatment strategies.

### 4.2. Optimization of VI-RADS Sequence and Diagnostic Accuracy of Biparametric MRI (bpMRI) VI-RADS

T2WI, DWI, and DCE are the key sequences of mpMRI. The scanning image should include the entire bladder, proximal urethra, and pelvic lymph nodes. The bladder is a hollow pelvic organ [83], and about one third of tumors originate from the trigone area, bladder neck area, and ureteral orifice [10]. More tumors come from the lateral wall, and conventional axial scans do not display the base of tumors in the neck area and posterior wall of the bladder well. Therefore, it is recommended to include at least two scan directions in the scan sequence to ensure that sections perpendicular to the tumor base are obtained [10]. However, if the scanning range or imaging plane is unreasonable, 2D T2WI does not allow retrospective post-processing, while 3D FSE acquisition can reconstruct any plane perpendicular to the tumor base according to the reader’s needs, which will help improve the efficiency of the examination and diagnostic performance [84]. It is easy to obtain multi-directional images in traditional T2WI and DCE sequences, but it is relatively difficult to obtain multi-directional images in DWI due to the limitations of scanning equipment. Meng et al. used reduced field of view DWI (rFOV DWI) and conventional single-plane DWI to image 61 patients with bladder cancer [85] and used the VI-RADS score to predict the possibility of muscle layer infiltration, and compared the advantages of the former. The results showed that VI-RADS based on biplanar rFOV DWI improved the ability to predict muscular invasion of bladder cancer (AUC = 0.946). Biplanar DWI scan may provide more diagnostic confidence than single-plane scans and significantly improve image quality, making it suitable for widespread application. The recommended b-value in the VI-RADS consensus ranges from 800 to 1000 s/mm^2^. Studies found that the best tumor contour was displayed when the b-value was 1500 s/mm^2^, and a better evaluation of the tumor–bladder wall interface could be obtained when the b-value was 1000 s/mm^2^ [86].

Several studies have shown that contrast-free MRI scanning protocols can be used for local staging of tumors, including rectal and prostate cancers, and have achieved high diagnostic accuracy [87,88]. Therefore, Delli Pizzi et al. first explored the accuracy of bpMRI in diagnosing muscular invasion of bladder cancer [89]. The study included 38 patients with bladder cancer, and four readers independently scored bpMRI and mpMRI images using VI-RADS to compare the diagnostic performance of the two study protocols. The results showed that the bpMRI protocol composed of T2WI and DWI had similar diagnostic performance for detecting muscular invasion compared to the mpMRI protocol. However, the number of cases included in this study was relatively limited. Aslan et al. validated the similar diagnostic performance of bpMRI and mpMRI in a larger sample (128 cases), and emphasized that DWI had high diagnostic performance (AUC = 0.947) in diagnosing muscular invasion when considering a single sequence [90]. However, DCE is also important in detecting muscular invasion in small tumors, and the diagnostic experience of the readers does not significantly affect the diagnostic performance. Watanabe et al. prospectively included 163 patients with bladder cancer to investigate the clinical effectiveness of non-contrast-enhanced VI-RADS (NCE-VI-RADS, T2WI + DWI) [91]. The study innovatively used denoising deep learning reconstruction (dDLR) of T2WI sequences. The results showed that the additional use of dDLR may further improve the accuracy of NCE-VI-RADS, which is almost identical to the diagnostic accuracy of traditional VI-RADS. However, the HGMRI scanner used in this study is not widely used at present, so further prospective and multi-institutional validation studies are needed.

In a meta-analysis involving 2344 patients from 17 studies, the diagnostic efficacy of bpMRI was analyzed in seven studies (1041 patients), and it was found that the diagnostic efficacy of bpMRI on muscular invasion was similar to mpMRI in pre-treatment MR images of patients [92]. There was no significant difference in sensitivity between the two when using a cutoff value of 3 or 4 for diagnosing muscular invasion, but bpMRI had higher specificity. However, most studies were retrospective or had small sample sizes and lacked prospective and large-sample research. Therefore, caution should be exercised when omitting DCE sequences in clinical practice. Similarly, some research results have shown that increasing the cutoff value to 3 improves the diagnostic efficacy of bpMRI and there is good inter-reader consistency [93].

Reader experience as well as tumor size and morphology have little effect on the accuracy of bpMRI. Meng et al. innovatively divided bladder cancer VI-RADS scores into consistent and inconsistent groups based on whether they conformed to the scoring system criteria. They described in detail the imaging characteristics of patients in the inconsistent group and emphasized the necessity of DCE sequences while also validating that bpMRI has similar diagnostic efficacy to mpMRI for muscular invasion, which was consistent with the conclusions of previous studies [94]. The application of NCE-VI-RADS avoids the use of contrast agent, reduces the examination costs, and shortens the examination time, and deserves further exploration and widespread use.

### 4.3. The Influence of Reader Experience on the Accuracy of VI-RADS and Consistency Analysis

The accuracy of VI-RADS in evaluating muscle invasion of bladder cancer has been recognized, but it is still a subjective scoring system. The impact of reader experience and inter-reader consistency on the system’s diagnostic performance needs to be further explored and validated.

Ueno et al. conducted the first in-depth study on the inter-reader consistency of VI-RADS, involving five radiologists with mpMRI scores. It was found that the intraclass correlation coefficient (ICC) among these five radiologists was 0.85, indicating excellent consistency, and the combined accuracy for diagnosing muscle invasion was high (AUC = 0.90) [73]. However, this study did not explore the diagnostic experience of readers and was a single-center study with a relatively small sample size (*n* = 74). Barchetti et al. included 78 patients with bladder cancer and two radiologists with special interest in urogenital imaging score VI-RADS independently [95]. These two radiologists had more than 10 years and 5 years of diagnostic experience, respectively, and scored three mpMRI sequences using VI-RADS separately to analyze inter-observer agreement. The results showed excellent agreement for T2WI as a single sequence, good to moderate agreement for DWI and DCE sequences, and overall good agreement for VI-RADS scores. Delli Pizzi et al. investigated the difference in diagnostic performance between bpMRI- and mpMRI-based VI-RADS by assigning different schemes to four abdominal imaging specialists with varying years of diagnostic experience (10 years, 15 years, 1 year, and 2 years) [89]. To avoid reading bias, there should have been at least a four-week interval between different schemes. The study found that among all four readers, regardless of their level of experience, there was no significant difference in diagnostic performance between the two different schemes. Among radiology residents, the bladder MRI and VI-RADS scoring interactive special education program significantly improved the diagnostic efficiency of readers over time, and the overall improvement was achieved after 100–150 cases, with good practicability and popularizations [96].

Some studies have demonstrated good consistency in radiologists’ diagnosis of VI-RADS [75], but readers’ acceptance of VI-RADS in daily clinical practice has not been evaluated. A multicenter study can further validate the accuracy of VI-RADS in assessing muscular invasion in bladder cancer and better verify consistency among readers. A total of 393 patients with bladder cancer at four hospitals were enrolled by Metwally et al., and their images were independently scored by four radiologists with 10, 12, 13, and 16 years of radiological diagnosis experience who used VI-RADS in their daily practice, and questionnaires were collected from diagnostic radiologists [97]. The results showed that the overall VI-RADS scores of the four readers had excellent consistency, and DCE sequence was moderate. T2WI and DWI scores were very good and good, and the questionnaire results showed that the readers believed that “structured reporting of bladder tumors should be encouraged” and “I will apply VI-RADS in routine clinical reports”. VI-RADS can accurately assess MIBC for both experienced and inexperienced readers with or without DCE, but there are significant differences among readers in the assessment of small lesions [98]. Jazayeri et al. reviewed the repeatability and diagnostic reliability of VI-RADS in detecting MIBC, and found that a total of 2439 patients with bladder cancer were included in 19 works in the literature [99]. VI-RADS was used to determine that the consistency of MIBC readers ranged from 0.45 to 0.96, and the final weighted average consistency was 0.76, indicating significant publication bias in readers’ reliability. There is good consistency among radiologists of different levels of expertise in the use of VI-RADS for bladder cancer staging.

The widespread application of VI-RADS is not limited to radiologists alone. It is crucial for urologists to be skilled in its use and interpretation for their management and treatment decisions for patients [100]. Arita et al. conducted a study on 66 untreated bladder cancer patients by using 3D FSE T2WI sequences for scanning [84]. The VI-RADS scores were completed by two radiologists and two urologists to assess inter-reader consistency. Although urologists had lower sensitivity and specificity in scoring the DCE sequence compared to radiologists, the observations showed good consistency. However, there was no significant difference in the overall accuracy of VI-RADS scoring. Therefore, NCE VI-RADS is also worthy of widespread application by urologists.

### 4.4. The Additional Value of Quantitative Imaging Parameters for VI-RADS

The diagnostic accuracy of VI-RADS for muscle invasion of bladder cancer is relatively high, but it is still in the stage of qualitative diagnosis. Therefore, some researchers have proposed to combine the existing quantitative diagnosis methods of bladder cancer with VI-RADS to obtain additional diagnostic value or further improve its diagnostic accuracy.

Li et al. obtained quantitative parameters by analyzing the whole-volume ADC histogram analysis of 80 cases of bladder tumors, realized the differential diagnosis of MIBC and NMIBC, and used DWI images to perform VI-RADS scoring on a single sequence [101]. The study combined quantitative parameters with DWI-VI-RADS to explore its combined diagnostic value. It was found that ADC parameters, skewness, and kurtosis in whole-volume histogram parameters had high diagnostic value in differentiating MIBC from NMIBC. In addition, combining skewness with DWI-VI-RADS can further improve its diagnostic performance. When the VI-RADS score was 3, the TCL truncation value was 19.5 mm, which could be used as an important quantitative parameter for determining muscle invasion. In addition to the histograms, radiomics has been widely and accurately applied in bladder cancer. Therefore, researchers have combined radiomic parameters with VI-RADS to further explore their additional diagnostic value [102].

Zheng et al. constructed a radiomics-clinical nomogram to differentiate high- from low-grade bladder cancer preoperatively [103]. The study included 185 patients with mpMRI-detected bladder cancer, and radiomic features were extracted from DCE and T2WI images. The radiomics-clinical nomogram combining radiomic features and VI-RADS had high diagnostic accuracy in both the training set (AUC = 0.956) and validation set (AUC = 0.958), indicating that the combination of radiomic features and VI-RADS further improved the diagnostic ability and assisted clinical decision making [104].

### 4.5. VI-RADS and Efficacy Evaluation of Bladder Cancer

The use of VI-RADS scoring is suitable for various situations. Currently, it is most appropriate before TURBt and intravesical chemotherapy [10]. Preoperative MRI and VI-RADS scoring can be used to predict treatment outcomes and help select the best method to treat the tumor. In high-risk NMIBC patients, VI-RADS scoring can be used for risk stratification of the disease and as an indication for tumor resection or avoiding secondary resection. As previously mentioned, bladder cancer has a high recurrence rate [105], and this method can be used as a follow-up diagnostic tool for disease monitoring. In the surveillance of NMIBC, MRI may serve as a reliable and non-invasive alternative to bladder cancer examination, reducing disease-related costs. In this case, structural changes in the bladder wall resulting from treatment must be considered. On T2W images, bladder wall thickening caused by fibrosis or inflammation secondary to surgery can be misdiagnosed as tumor recurrence or residue. To overcome these issues, the application of DWI has been proven reliable in distinguishing between postoperative changes and malignant bladder tumors, and has higher efficacy than DCE.

The initial VI-RADS consensus recommends performing MRI at least 2 weeks after administration of TURBt and Bacillus Calmette–Guérin (BCG) to avoid overstaging due to post-treatment structural changes [106]. Additionally, MRI should be performed at least 2 days after cystoscopy or removal of urinary catheterization to reduce the risk of artifacts occurring. For patients with MIBC, MRI and VI-RADS scores can help predict which tumor patients will benefit from neoadjuvant chemotherapy, providing new insights into patient management while proposing the use of dual-parameter examinations to optimize imaging methods [107].

Pecoraro et al. explored the feasibility of a new classification scoring method, NAC-VI-RADS, for radiologic assessment of response (RaR) to determine the treatment response spectrum in MIBC patients [108]. Unlike conventional VI-RADS evaluation criteria, NAC-VI-RADS provides response assessment criteria (including tumor activity and efficacy), with no bladder lesions marked as complete RaR (NAC-VI-RADS 1-2), any radiological downgrading with evidence of residual tumor marked as partial RaR (NAC-VI-RADS 3-4), and lack of radiological downgrading or upgrading marked as no RaR (NAC-VI-RADS 5). The study findings demonstrated that NAC-VI-RADS was able to match all final radical cystectomy pathology results, proving the feasibility of this scoring system and potentially transforming the way MIBC survival risk is assessed.

MRI and VI-RADS scores have been used as clinical predictive indicators for undergoing radical cystectomy in patients with locally advanced bladder cancer. Giudice et al. evaluated the diagnostic accuracy of VI-RADS in distinguishing features of stage II and III bladder cancer and found that overall accuracy was higher not only in relation to pathologically confirmed ≥ pT3 tumors (AUC 94.2%, 95% CI: 88.7–99.7), and a VI-RADS score of 5 was an independent predictor significantly delaying time-to-cystectomy (TTC) [109]. VI-RADS can be used to reduce the reliance on deep resection for MIBC pathology and to safely and accurately select patients for histological sampling prior to final treatment.

At present, the MRI studies on bladder cancer mainly focus on the field of VI-RADS. Researchers have not only verified the diagnostic accuracy of VI-RADS but also further optimized, supplemented, and made suggestions for the existing scoring system, and improved its diagnostic value through the supplementary application of a series of quantitative parameters to achieve more accurate preoperative staging diagnosis, risk assessment, and prognosis assessment. In order to better apply scientific research results to clinical practice, simple and accurate quantitative parameters and characteristics offer more application prospects.

## 5. Application of Other MRI Functional Sequences in Bladder Cancer

T2*-weighted imaging is a technique to calculate the apparent transverse relaxation rate R2* (R2* = 1/T2*) by using multiple gradient echo sequences to fit the attenuation curves of multiple echo images into a single exponential model. Studies have shown that R2* values are negatively related to tissue oxygen pressure, reflecting tissue paramagnetic characteristics such as deoxyhemoglobin presence, which has led to its application in various types of tumors [110].

Wang et al. applied T2*-weighted imaging to the study of bladder cancer for the first time, and used postoperative histopathological results as a reference standard to evaluate the staging and grading of bladder cancer [111]. Their findings revealed significant differences in both R^’*’and ADC values between low-grade and high-grade bladder cancers. By utilizing R^’*’, they were able to distinguish between high- and low-grade bladder cancers as well as NMIBC and MIBC cases, with a diagnostic AUC of 0.714 and 0.682, respectively, providing additional quantitative parameters in addition to ADC for diagnosing tumor grade classification or stage determination in patients with bladder cancer.

Amide proton transfer (APT) imaging is an emerging magnetic resonance technique based on chemical exchange saturation transfer (CEST), which is sensitive to mobile proteins and peptides in tissues [112]. APT imaging selectively saturates the amide protons (-NH) in endogenous cytoplasmic proteins and peptide bonds using a frequency-selective saturation pulse at 3.5 ppm, and this saturation is transferred to water protons through proton exchange, making it sensitive to the spatial distribution of mobile proteins and peptides. Currently, APT imaging is widely used for tumor detection, differentiation, grading, and evaluation of chemotherapy efficacy but has limited applications in bladder cancer [113]. Wang et al., by including 11 healthy volunteers and 18 bladder cancer patients, first optimized the imaging parameters through in vitro model experiments [114]. The optimal scanning scheme parameters were found to be one excitation number, a saturation power of 2 mT, and a saturation time of 2 s. MTRasym showed good consistency among all subjects, with significantly higher values observed in bladder cancer tissue compared to normal bladder wall. The study confirmed the feasibility of applying APT imaging to bladder cancer assessment and its potential for evaluating tumor grading and muscle invasion. However, this study represented preliminary exploratory research with a small sample size of bladder cancer cases and lacked the assessment of the accuracy of APT for staging or grading bladder cancer. Wang et al., by including 83 bladder cancer patients (51 high-grade and 32 low-grade cases), investigated the potential of APT imaging for assessing tumor grading in addition to evaluating its added value compared to ADC [115]. The results showed that high-grade bladder cancers had higher APTw values and lower ADC values, and both measures had similar diagnostic performance for distinguishing high- from low-grade tumors (AUC = 0.84, *p* = 0.94). Combining both measures further improved diagnostic performance (AUC = 0.93, *p* = 0.01). APT imaging provided valuable information regarding histological grading of bladder cancer while also providing additional value beyond ADC measurements. However, these conclusions still require further prospective validation from multicenter studies with larger sample sizes.

## 6. The Role of Machine Learning and Artificial Intelligence (AI) Based on MRI Radiomics in Bladder Cancer

With advancements in databases and algorithms, AI has found widespread applications in various fields, including medicine. AI holds great promise in improving the accuracy of diagnosis and predicting the prognosis of bladder cancer. Automated machine learning techniques based on radiomics features derived from MRI have been utilized in bladder cancer diagnosis and have demonstrated promising potential for practical implementation [116].

Huang et al. developed an automated machine learning (AutoML) model based on MR radiomic features, semantic features, and clinical features from T2WI, DWI, and DCE images [117]. This AutoML model can identify MIBC patients with variant histology, with high diagnostic efficiency in both the training set (AUC = 0.955) and test set (AUC = 0.932), which provides a non-invasive and low-cost preoperative prediction tool for clinical decision making. Chen et al. conducted a study enrolling 445 patients with bladder cancer, and developed a full-sequence MRI model for preoperative prediction of the depth of bladder cancer infiltration, based on T1-weighted images, T2WI, DWI, and DCE images, with excellent accuracy (AUC = 0.857) [118]. This model was derived from six advanced machine learning techniques, including Least Absolute Shrinkage and Selection Operator (LASSO) and Max Relevance Min Redundancy (mRMR). Fan et al. performed a retrospective study enrolling 91 MIBC patients and analyzed DNA sequencing data, prognostic information, and radiomics features from T2WI and DWI [119]. Radiomics signatures were extracted to construct a nomogram based on logistic regression for predicting the stratification in the training cohort, using LASSO. Five radiomics signatures were identified as being related to risk stratification and the nomogram achieved an accuracy of 0.889 and an ROC curve of 0.909, which means it can serve as a prediction tool for stratification and clinical decision making.

However, there are still many challenges to overcome in clinical practice, including standardization of MR images, data collection and analysis, interpretability of AI models or nomograms, validation of patient cohorts, and legal and ethical implications. Despite these challenges, AI has been proven to have great potential for improving BC management, such as by identifying novel biomarkers and therapeutic targets. To achieve this goal, more research is still needed.

## 7. Conclusions

MpMRI is a promising tool widely used for preoperative staging evaluation of bladder cancer. However, surgical pathology remains the reference standard for diagnosis and staging. The application of preoperative MRI can help expedite clinical pathway management. VI-RADS scoring has proven to be a reliable tool for local staging of bladder cancer with high accuracy in preoperative staging. However, further research is needed to strengthen its predictive value for tumor invasiveness and treatment efficacy assessment. Future work should focus on conducting more prospective, multicenter studies to validate the additional value of quantitative studies and optimize prediction models. Although significant progress has been made in bladder cancer imaging research, from basic tumor identification and differentiation to precise tumor staging and grading, relying solely on disease diagnosis is no longer sufficient to meet clinical requirements. In the future, there will be an increasing shift towards utilizing imaging for precise individualized treatment and management.

## Data Availability

Not applicable.
